# Outcome of Pulmonary Embolism with and without Ischemic Stroke

**DOI:** 10.3390/jcm13102730

**Published:** 2024-05-07

**Authors:** Karsten Keller, Volker H. Schmitt, Omar Hahad, Lukas Hobohm

**Affiliations:** 1 Department of Cardiology, University Medical Center of the Johannes Gutenberg-University Mainz, 55131 Mainz, Germany; volker.schmitt@unimedizin-mainz.de (V.H.S.); omar.hahad@unimedizin-mainz.de (O.H.); lukas.hobohm@unimedizin-mainz.de (L.H.); 2 Center for Thrombosis and Hemostasis (CTH), University Medical Center of the Johannes Gutenberg-University Mainz, 55131 Mainz, Germany; 3 Medical Clinic VII, Department of Sports Medicine, University Hospital Heidelberg, 69120 Heidelberg, Germany; 4 German Center for Cardiovascular Research (DZHK), Partner Site Rhine Main, 55131 Mainz, Germany

**Keywords:** systemic thrombolysis, mortality, ischemic stroke, pulmonary embolism

## Abstract

**Background:** Ischemic stroke is the second, and pulmonary embolism (PE) is the third most common cardiovascular cause of death after myocardial infarction. Data regarding risk factors for ischemic stroke in patients with acute PE are limited. **Methods:** Patients were selected by screening the German nationwide in-patient sample for PE (ICD-code I26) and were stratified by ischemic stroke (ICD code I63) and compared. **Results:** The nationwide in-patient sample comprised 346,586 hospitalized PE patients (53.3% females) in Germany from 2011 to 2014; among these, 6704 (1.9%) patients had additionally an ischemic stroke. PE patients with ischemic stroke had a higher in-hospital mortality rate than those without (28.9% vs. 14.5%, *p* < 0.001). Ischemic stroke was independently associated with in-hospital death (OR 2.424, 95%CI 2.278–2.579, *p* < 0.001). Deep venous thrombosis and/or thrombophlebitis (DVT) combined with heart septal defect (OR 24.714 [95%CI 20.693–29.517], *p* < 0.001) as well as atrial fibrillation/flutter (OR 2.060 [95%CI 1.943–2.183], *p* < 0.001) were independent risk factors for stroke in PE patients. Systemic thrombolysis was associated with a better survival in PE patients with ischemic thrombolysis who underwent cardio-pulmonary resuscitation (CPR, OR 0.55 [95%CI 0.36–0.84], *p* = 0.006). **Conclusions:** Ischemic stroke did negatively affect the survival of PE. Combination of DVT and heart septal defect and atrial fibrillation/flutter were strong and independent risk factors for ischemic stroke in PE patients. In PE patients with ischemic stroke, who had to underwent CPR, systemic thrombolysis was associated with improved survival.

## 1. Introduction

Pulmonary embolism (PE) and ischemic stroke are acute and life-threatening cardiovascular emergency events related to high morbidity and mortality [1,2,3,4,5,6,7,8]. While the annual incidence of PE inclined in the past decades to 109 per 100,000 in Germany during the year 2015 in the light of decreasing case fatality [3,9,10,11], the absolute annual numbers of incident strokes increased by 70% from 1990 to 2019, and the global age-standardized incidence rate of ischemic stroke is projected to increase to 89 per 100,000 population by 2030 worldwide [12,13,14].

PE is caused by an acute embolization in the pulmonary artery bed affecting pulmonary circulation driven by the vascular occlusion, which impairs gas exchange and circulation properties [3,11], whereas in ischemic stroke, the occlusion of an intracranial or neck blood vessel is the initiating event that in most cases impairs blood flow to a portion of the brain, resulting in infarction of brain tissue [15,16]. Ischemic stroke is the second, and PE is the third most common cardiovascular cause of death after myocardial infarction [3,10,16,17]. Mortality caused by PE is closely related to patients’ hemodynamic status, cardiac involvement, including right ventricular dysfunction (RVD) and/or myocardial injury, as well as patients’ individual comorbidity profile [2,3,4,6,18,19,20,21,22,23,24]. In ischemic stroke, coma and cerebral edema, as well as heart failure and advanced age, appeared to be associated with poor short-term outcomes [25,26].

In this context, it is important to note that PE-related deaths account for approximately 20–25% of the early deaths in stroke patients [27]. Without prophylaxis for venous thromboembolism (VTE), approximately 75% of the stroke patients with hemiplegia might develop a deep venous thrombosis and 20% of these patients an acute PE, which is fatal in 1–2% of the patients [27]. Notably, the risk of the occurrence of deep venous thrombosis is strongly related to the degree of paralysis with predilection in the paralyzed leg [28,29]. It is well known that approximately half of clinically evident PE events following stroke manifest as sudden death [30,31] and the majority of these patients had no clinical evidence of deep venous thrombosis [7,30]. As aforementioned, both cardiovascular diseases (PE and ischemic stroke) are life-threatening events if they occur singularly without influence of the other entity and share in part the pathomechanism of a blot clot occlude an artery to an essential vital organ. It has to be expected that a combination of both acute events compromising the perfusion of two vital essential organs is accompanied by a largely aggravated risk of death. Although studies report an incidence of acute ischemic stroke after a PE ranging between 1 and 10%, concomitant ischemic stroke directly after acute PE in the acute phase of PE is infrequent [32]. Data regarding the impact of ischemic stroke on the outcome of acute PE and risk factors for ischemic stroke in patients with acute PE are very sparse [32,33]. Thus, the objective of our study was to identify risk factors for ischemic stroke in PE patients, evaluate the influence of ischemic stroke on the survival of PE patients, and also to investigate the effect of systemic thrombolysis on survival in PE patients with a co-prevalence of PE and ischemic stroke.

## 2. Methods and Patients

For our analysis, we selected all in-patients diagnosed with acute PE (ICD code I26) in the German nationwide in-patient sample stratified for ischemic stroke (ICD code I63) during the years 2011 and 2014. In Germany, patients’ diagnoses must be coded from all hospitals according to ICD-10-GM (International Classification of Diseases, 10th Revision with German Modification) to obtain remuneration for the rendered services/efforts, whereas surgical, diagnostical, and/or interventional procedures have to be coded according to OPS codes (Operationen- und Prozedurenschlüssel). DRG diagnoses of in-patients are collected and evaluated by the Federal Statistical Office of Germany (Statistisches Bundesamt). PE patients of all ages were included in the analysis without exclusion (source: RDC of the Federal Statistical Office and the Statistical Offices of the Federal States, DRG Statistics 2011–2014, own calculations). Clinical patient characteristics of each patient are also coded in the German nationwide in-patient sample and were assessed by us. Thus, these data allow us to characterize the comorbid burden of the patients (Table 1 illustrates the patient characteristics).

### 2.1. Study Outcomes

The primary outcome of this study was all-cause death during in-hospital stay (in-hospital death). We focused on in-hospital survival as a primary outcome since most treatment studies use the case-fatality rate singularly as an important central outcome. Especially regarding the benefit of the reperfusion treatment of systemic lysis in these patients, all-cause death during in-hospital stay (in-hospital death) was assessed as the key outcome parameter. The secondary outcome was defined as the combined adverse in-hospital outcome (comprising all-cause in-hospital death, mechanical ventilation, or cardio-pulmonary resuscitation), as most risk stratification studies use such a combined outcome to prove risk stratification markers. The overlapping of the primary and secondary outcomes is necessary because of the different foci of these outcomes to generate a comparability to previously published study results.

### 2.2. Ethical Aspects and Study Oversight

Because this study did not involve direct access by the investigators (us) to data on individual patients but only access to summary results provided by the Research Data Center, approval by an ethics committee and informed consent were not required, in accordance with German law.

#### 2.2.1. Definitions

Adverse in-hospital events were defined as a composite of all-cause in-hospital death, the need for mechanical ventilation, and/or cardio-pulmonary resuscitation (CPR). Chronic lung diseases comprised bronchial asthma, chronic obstructive lung disease, pulmonary arterial hypertension, and interstitial lung diseases. Renal insufficiency included diagnosis of all renal insufficiency stages. Coagulation abnormalities comprised coagulopathies, hemophilia, purpura, and bleeding diathesis consisting of disseminated intravascular coagulation.

#### 2.2.2. Statistics

Descriptive statistics for the relevant comparisons regarding the patients’ characteristics of 1st) PE with additional ischemic stroke vs. PE without ischemic stroke as well as 2nd) in PE patients with additional stroke comparing survivors vs. non-survivors were provided with median and interquartile range (IQR), or absolute numbers and corresponding percentages. Continuous variables were compared using the Wilcoxon–Whitney U test and categorical variables with Fisher’s exact or chi^2^ test, as appropriate.

Univariate and multivariate logistic regression models were calculated to identify (I) risk factors for ischemic stroke in PE patients, (II) predictors for in-hospital case-fatality in patients suffering from both PE and ischemic stroke, and (III) the impact of systemic thrombolysis on the survival of PE patients with additional ischemic stroke. We calculated the multivariate regression models with different adjustments to examine whether the significant associations, which were detected in univariate regressions, remained significant after adjustment for the additional parameters. The multivariate regressions were calculated with all the parameters in one model. Results were presented as odds ratios (OR) and 95% confidence intervals (CI).

We calculated Cox regression models for survival analysis related to in-hospital stay, comparing (I) PE patients with ischemic stroke vs. those without ischemic stroke and (II) PE patients with additional ischemic stroke, PE patients with co-prevalence of ischemic stroke stratified for atrial fibrillation/flutter (AF).

In PE patients with ischemic stroke aged ≥ 18 years (after exclusion of those patients with surgical thrombectomy), we compared the in-hospital mortality between the subgroups with and without treatment of systemic thrombolysis in all patients as well as in those patients with additional CPR only.

The software SPSS^®^ (version 20.0; SPSS Inc., Chicago, IL, USA) was used for the computerized analysis. Only *p*-values of <0.05 (two-sided) were statistically significant.

## 3. Results

The German nationwide in-patient sample comprised 346,586 hospitalized patients (53.3% females) with acute PE in Germany in the years 2011–2014. Among these, 6704 (1.9%) patients were additionally diagnosed with ischemic stroke. In total, 51,296 PE patients died during hospitalization (14.8%). The case-fatality rate was higher in PE patients with ischemic stroke than in those without ischemic stroke (28.9% vs. 14.5%, *p* < 0.001) (Table 1).

**Table 1:** Patients’ characteristics, medical history, presentation, treatments, and adverse in-hospital events of the 346,586 PE patients (ICD I26) stratified according to additional diagnosis of ischemic stroke (ICD I63).

Parameters	PE Patients with Ischemic Stroke (n = 6704; 1.9%)	PE Patients without Ischemic Stroke (n = 339,882; 98.1%)	*p*-Value
Age	73.0 (63.0–81.0)	72.0 (60.0–80.0)	<0.001
Age ≥ 18 years	6698 (99.9%)	339,191 (99.8%)	0.038
Gender (Females)	3598 (53.7%)	181,201 (53.3%)	0.564
In-hospital stay (days)	18 (9–29)	6 (9–15)	<0.001
Obesity	579 (8.6%)	29,349 (8.6%)	0.996
**VTE risk factors**			
Surgery during in-hospital stay	4832 (72.1%)	172,485 (50.7%)	<0.001
Cancer	1113 (16.6%)	68,017 (20.0%)	<0.001
**Comorbidities**			
Chronic (left) heart failure	1597 (23.8%)	74,067 (21.8%)	<0.001
Chronic lung disease	1516 (22.6%)	64,830 (19.1%)	<0.001
Arterial hypertension	3627 (54.1%)	152,073 (44.7%)	<0.001
Renal insufficiency	1642 (24.5%)	73,163 (21.5%)	<0.001
Diabetes mellitus (type 2 diabetes)	1710 (25.5%)	63,657 (18.7%)	<0.001
Deep venous thrombosis or thrombophlebitis	1605 (23.9%)	124,872 (36.7%)	<0.001
Coagulation abnormalities	1054 (15.7%)	32,319 (9.5%)	<0.001
Heart septal defect	534 (8.0%)	1178 (0.3%)	<0.001
Atrial fibrillation/flutter	2067 (30.8%)	51,026 (15.0%)	<0.001
Combination of DVT and heart septal defect	221 (3.3%)	385 (0.1%)	<0.001
**Risk stratification**			
Tachycardia	226 (3.4%)	6144 (1.8%)	<0.001
Syncope	112 (1.7%)	7838 (2.3%)	0.001
Shock	506 (7.5%)	13,449 (4.0%)	<0.001
Myocardial Injury	393 (5.9%)	7549 (2.2%)	<0.001
RV dysfunction	1991 (29.7%)	92,812 (27.3%)	<0.001
**Adverse events and treatments during the in-hospital stay after acute PE**			
Adverse in-hospital outcome (comprising all-cause in-hospital death, mechanical ventilation, or cardio-pulmonary resuscitation)	3045 (45.4%)	69,556 (20.5%)	<0.001
Cardio-pulmonary resuscitation	731 (10.9%)	22,313 (6.6%)	<0.001
Mechanical ventilation	1876 (28.0%)	37,982 (11.2%)	<0.001
All cause in-hospital death	1938 (28.9%)	49,358 (14.5%)	<0.001
Systemic thrombolysis	795 (11.9%)	14,740 (4.3%)	<0.001
Surgical embolectomy of PE thrombus	18 (0.3%)	468 (0.1%)	0.005
Transfusion of blood constituents	1467 (21.9%)	39,479 (11.6%)	<0.001

Abbreviations: VTE indicates venous thromboembolism, which comprises the two VTE entities of pulmonary embolism and deep venous thrombosis; RV, right ventricular. *p*-values of <0.05 (two-sided) mark statistically significant values.

### 3.1. Comparison of the Characteristics of PE Patients with and without Ischemic Stroke

PE patients with ischemic stroke were older and underwent more surgery during in-hospital stays compared to those without ischemic stroke (Table 1). In contrast, cancer was more frequently found in PE patients without stroke. All investigated relevant comorbidities such as chronic heart and renal failure, as well as chronic lung disease and AF, were more prevalent in PE patients with ischemic stroke. Noteworthy, coagulation abnormalities were distinctly more often detected in PE patients with ischemic stroke than without (15.7% vs. 9.5%, *p* < 0.001) (Table 1). In summary, regarding typical VTE risk factors, surgery, coagulation abnormalities, and heart failure, but not cancer, were more often seen in PE patients with ischemic stroke. Heart septal defects with and without detected deep venous thrombosis and/or thrombophlebitis (DVT)—as a possible cause of paradoxical embolism—were more often identified in PE patients with ischemic stroke.

PE patients with ischemic stroke showed more frequently signs of hemodynamic compromise, such as RVD, tachycardia, myocardial injury, as well as shock, whereas syncope was less common in these patients.

**Figure 1:**
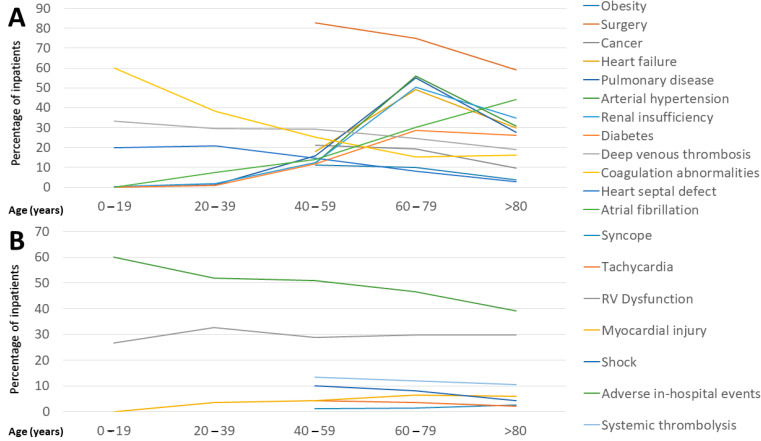
Risk factors, comorbidities, risk stratification markers, systemic thrombolysis, and adverse events in stroke with additionally diagnosed PE stratified by age groups. (**A**) Risk factors and comorbidities stratified by age groups. (**B**) Risk stratification markers, systemic thrombolysis, and adverse events stratified by age groups.

While the proportion of hereditary factors such as coagulation abnormalities and heart septal defects decreased over the time, the prevalence of established cardiovascular risk factors and comorbidities increased with growing age, as expected (Figure 1A).

PE patients with ischemic stroke exhibited a risk ratio (RR) of 2.2 times for the secondary outcome (combined adverse in-hospital outcome), were 1.7 times more likely to undergo CPR, had a prolonged in-hospital stay, and faced a 2.0-fold increased risk for in-hospital case-fatality in comparison to PE patients without ischemic stroke (primary outcome of the study). Systemic thrombolysis was 2.8-fold (RR) more often administered in PE patients with ischemic stroke compared to those without (Table 1). The percentage of adverse events during the in-hospital phase decreased markedly with increasing age (Figure 1B). PE patients with ischemic stroke had a more than 24-fold prevalence of heart septal defect and a more than 2-fold prevalence of AF, independently of other factors (Table 2). The combination of DVT and heart septal defect was independently associated with ischemic stroke in PE patients (OR 24.714 [95%CI 20.693–29.517], *p* < 0.001). Increasing age (OR 1.005 [95%CI 1.003–1.007], *p* < 0.001), surgery (OR 1.686 [95%CI 1.590–1.789], *p* < 0.001), arterial hypertension (OR 1.259 [95%CI 1.196–1.325], *p* < 0.001), diabetes mellitus type 2 (OR 1.233 [95%CI 1.114–1.364], *p* < 0.001), as well as AF (OR 2.060 [95%CI 1.943–2.183], *p* < 0.001) were independent risk factors for ischemic stroke in PE patients (Table 2).

PE patients with ischemic stroke events had a 2.4-fold higher risk of dying during their in-hospital stay in comparison to acute PE patients without ischemic stroke (univariate analysis: OR 2.393, 95%CI 2.268–2.525, *p* < 0.001). This result remained stable after adjustment for age and sex (OR 2.335, 95%CI 2.211–2.466, *p* < 0.001) as well as additional adjustment for the comorbidities/conditions of obesity, surgery, cancer, heart failure, chronic lung disease, arterial hypertension, renal insufficiency, diabetes mellitus type 2, DVT, coagulation abnormalities, heart septal defect, AF, tachycardia, shock, myocardial injury, and RVD (OR 2.424, 95%CI 2.278–2.579, *p* < 0.001).

**Figure 2:**
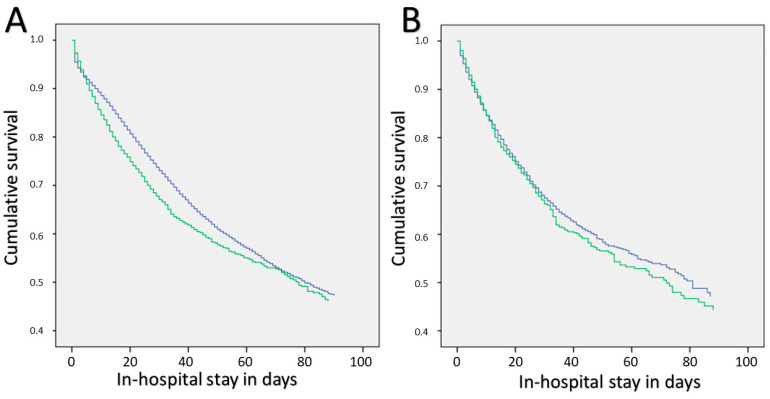
(**A**) Kaplan–Meier plot for the in-hospital mortality of PE patients with additionally coded ischemic stroke (green line) and without (blue line) related to the in-hospital stay. (**B**) Kaplan–Meier plot for the in-hospital mortality of PE patients with additionally diagnosed ischemic stroke stratified by the comorbidity of atrial fibrillation/flutter related to the in-hospital stay (blue line: patients without AF; green line: patients with AF).

In the crude Cox regression model, ischemic stroke in PE patients had a significant influence on in-hospital survival (HR 1.218, 95%CI 1.164–1.275, *p* < 0.001) related to the days of in-hospital stay. After adjustment for age and gender (HR 1.221, 95%CI 1.166–1.278, *p* < 0.001) as well as for age, gender, and the comorbidities/conditions of obesity, surgery, cancer, heart failure, chronic lung disease, arterial hypertension, renal insufficiency, diabetes mellitus type 2, DVT, coagulation abnormalities, heart septal defect, AF, tachycardia, shock, myocardial injury, and RVD (HR 1.313, 95%CI 1.254–1.375, *p* < 0.001), the result remained stable (Figure 2).

### 3.2. Predictors of In-Hospital Death in PE Patients with Ischemic Stroke

Results about the comparison of deceased PE patients with ischemic stroke vs. PE patients with ischemic stroke, who survived, showed that non-survivors were, on average, 2 years older, less often obese, but had more often chronic diseases such as chronic heart and renal failure, chronic lung diseases, cancer, and coagulation abnormalities (Table 3). Surgery as a typical VTE risk factor was less prevalent in non-survivors. While AF as a source of cardioembolic stroke was more often diagnosed in deceased PE patients with ischemic stroke, heart septal defect as well as DVT as a cause of paradoxical embolism were both singularly as well as in co-prevalence less frequently observed in non-survivors. Remarkably, patients with arterial hypertension showed a lower case-fatality rate (Table 3).

As expected, markers corresponding to hemodynamic compromise, such as tachycardia, RVD, myocardial injury, and shock, were all more prevalent in non-survivors (Table 3).

Independent predictors for in-hospital case-fatality in the group of PE patients with ischemic stroke, which were calculated with an OR of >1.5, were cancer, renal insufficiency, coagulation abnormalities, shock, and myocardial injury and RVD (Table 4). Remarkably, cardio-embolic stroke as ischemic stroke in PE patients with additional AF is also a strong and independent predictor for in-hospital case-fatality (OR 1.238 [95%CI 1.083–1.414], *p* = 0.002) (Table 4).

PE patients with ischemic stroke with an additional AF had a poorer prognosis with a higher case-fatality rate (HR 1.342, 95%CI 1.314–1.369, *p* < 0.001) related to the in-hospital stay in the computed Cox regression model. After adjustment for age and gender (HR 1.113, 95%CI 1.090–1.136, *p* < 0.001), the result remained stable, but not after additional adjustment for the comorbidities/conditions of obesity, surgery, cancer, heart failure, chronic lung disease, arterial hypertension, renal insufficiency, diabetes mellitus type 2, DVT, coagulation abnormalities, heart septal defect, tachycardia, shock, myocardial injury, and RVD (HR 0.998, 95%CI 0.976–1.020, *p* = 0.836) (Figure 2). Female sex, obesity, surgery during in-hospital stay, arterial hypertension, and DVT in combination with a heart septal defect were accompanied by a higher chance of survival (Table 3).

### 3.3. The Impact of Systemic Thrombolysis on In-Hospital Case-Fatality Rate

When focusing on 6680 PE patients with ischemic stroke aged ≥ 18 years (and after exclusion of patients with surgical thrombectomy), only 792 (11.9%) patients were treated with systemic thrombolysis. The in-hospital case-fatality rate was higher in patients treated with systemic thrombolysis compared to those without this treatment (35.5% vs. 27.9%, *p* < 0.001) when the analysis was performed for all patients regardless of hemodynamic compromise. This finding was confirmed in the logistic regression (OR 1.418, 95%CI 1.213–1.658, *p* < 0.001) in an univariate regression model, but interestingly, after adjustment for age, gender, in-hospital stay, obesity, surgery during hospitalization, cancer, heart failure, chronic lung disease, arterial hypertension, renal insufficiency, diabetes mellitus type 2, rheumatic diseases, DVT, tachycardia, shock, RVD, myocardial injury, coagulation abnormalities, and heart septal defect (OR 1.112, 95%CI 0.925–1.336, *p* = 0.259), this result was no longer significant.

In contrast, when narrowing the focus on the subgroup of 723 PE patients with ischemic stroke aged ≥ 18 years (excluding patients with surgical embolectomy) who underwent CPR, overall 226 (31.3%) were treated with systemic thrombolysis. The in-hospital case-fatality rate was slightly lower in patients treated with systemic thrombolysis compared to those without this treatment (66.4% vs. 69.8%, *p* = 0.354). Although systemic thrombolysis was not associated with a beneficial impact on survival in the univariate regression (OR 0.853, 95%CI 0.610–1.194, *p* = 0.354), after adjustment for age, gender, length of in-hospital stay, obesity, surgery during hospitalization, cancer, heart failure, chronic lung disease, arterial hypertension, renal insufficiency, diabetes mellitus type 2, rheumatic diseases, DVT, tachycardia, shock, RVD, myocardial injury, coagulation abnormalities, and heart septal defect, systemic thrombolysis was associated with higher survival in this crucial patient group (OR 0.548, 95%CI 0.358–0.840, *p* = 0.006).

## 4. Discussion

While VTE events are frequent and harmful complications in stroke patients [27,28,29,30,31,34] and the number of PE-related deaths may be underestimated since approximately half of clinically evident PE events following stroke present as sudden death [30,31], studies about the co-prevalence of ischemic stroke and PE are very limited [7,33].

The main results of our study can be summarized as follows: (I) Ischemic stroke negatively affected the survival of patients with acute PE. (II) Combination of DVT and heart septal defect, older age, surgery, arterial hypertension, diabetes mellitus type 2, as well as AF were independent risk factors for stroke in PE patients. (III) Age, cancer, chronic lung diseases, renal insufficiency, coagulation abnormalities, AF, and diabetes mellitus type 2 were independent risk factors for increased in-hospital case-fatality. (IV) Markers of hemodynamic compromise, such as shock, myocardial injury, and RVD, identified PE patients who were at increased risk of dying regardless of the diagnosis of stroke. (V) Systemic thrombolysis was used 2.8-fold more often in PE patients with ischemic stroke compared to those without stroke. (VI) The usage of systemic thrombolysis was accompanied by a survival benefit in PE patients with ischemic stroke who underwent CPR.

Ischemic stroke is the second, and PE is the third most common cardiovascular cause of death after myocardial infarction [3,10,16,17]. In this present study, PE patients showed an in-hospital mortality rate of 14.8%, which is in accordance with previously published studies [3,35]. When PE and ischemic stroke occurred both during the in-hospital course, the in-hospital case-fatality rate increased to 28.9%. Thus, the case-fatality rate was approximately doubled if both vascular events occurred in comparison to the case-fatality of PE without ischemic stroke and was approximately 3.5-fold increased in comparison to the reported in-hospital mortality rate of ischemic stroke patients in Germany, which ranges in these studies approximately between 5% and 7% [7,36,37]. PE patients’ risk of dying was 2.4-fold and therefore substantially increased by the occurrence of ischemic stroke, independently of age, sex, comorbidities, and signs of hemodynamic compromise. This finding was in line with other studies, showing that ischemic stroke complicates the outcome of different acute cardiovascular events [38,39].

Age, cancer, chronic lung diseases, renal insufficiency, coagulation abnormalities, AF, and diabetes mellitus type 2 were independent predictors of increased in-hospital case-fatality. PE patients with ischemic stroke revealed a higher burden of atherosclerotic diseases, chronic heart failure, and AF as possible sources of (cardioembolic) ischemic stroke events. It is well known that AF, especially, increases the risk of stroke [40,41,42,43,44]. In general, 15–30% of all strokes are due to AF, and this rate may even be underestimated since strokes caused by AF due to unidentified “silent” or paroxysmal AF remain frequently unrecognized [42,45,46,47,48,49]. In consensus with these reported percentages, we elucidated an AF rate of 31% in PE patients with additional strokes. Thromboembolic strokes due to AF are frequently devasting, accompanied by severe impairments or death [48,50,51]. In addition, cardiovascular risk factors are known risk factors for ischemic stroke [52]. In accordance with the literature, persistent foramen ovale, prolonged immobility, and hypercoagulable states are common risk factors for ischemic stroke in PE [32]. Paradoxical embolism might be one important cause of ‘cryptogenic’ ischemic stroke [53]. In accordance with literature [53,54], paradoxical embolism seems not to be a rare entity in ischemic stroke: in 8% of the ischemic stroke patients of our study, a heart septal defect was documented. In addition, our study results lead to the assumption that a paradoxical embolism is accompanied by a lower mortality rate, maybe due to a lower thrombus burden traveling through an existing heart septal defect to the brain. The important question in this context is whether the presence of a heart septum defect represents an association by chance or a true cause-and-effect relationship [53]. However, Konstantinides et al. [55] reported that a patent foramen ovale signifies a high risk of death and arterial thromboembolic complications, including ischemic stroke, in PE patients [55].

In view of the heterogeneity of the PE patients with additionally diagnosed ischemic stroke, we consider that the underlying pathomechanism of ischemic stroke has an important impact on the risk of dying during in-hospital stay. The following two pathomechanisms have to be distinguished: (I) paradoxical stroke: DVT leading to both PE and through heart septal defect to ischemic stroke [55]. (II) cardioembolic stroke and PE: emboli develop within the cardiac cavities, causing ischemic stroke as well as PE most frequently in the presence of AF [41,42,43,44]. The thrombotic material of PE has, in the majority of events, its origin in the deep veins, forming a DVT [56], but maybe it is also caused by AF, with the formation of the right heart thrombi [57,58]. (III) Atherosclerotic stroke in temporal context with PE in terms of PE as a stress factor in cerebral perfusion (hypotension due to PE) [56]. (IV) Presumably, the most frequent cause is the development of PE as a consequence of ischemic stroke, driven by typical VTE risk factors such as immobilization in stroke patients [27,28,29]. These differences regarding the pathomechanism have to be kept in mind when interpreting the data.

Our data confirm that the risk stratification markers shock, myocardial injury, and RVD work adequately in PE patients regardless of the presence of stroke to identify those PE patients who are at an increased risk of dying.

As expected, higher age and chronic diseases such as chronic heart and renal failure and chronic lung diseases, as well as cancer and coagulation abnormalities, were accompanied by a higher mortality rate in PE patients with ischemic stroke in accordance with previous PE [59] and ischemic stroke studies [60]. Our study results demonstrated an outstanding role of AF (frequently accompanied by severe impairment or death [48,50,51]) increasing the in-hospital mortality rate independently of other factors.

Of particular interest are factors that were accompanied by better survival. Although most studies reported a similar short-term survival between men and women in ischemic stroke patients [61,62,63,64], our data revealed a better survival in women with additionally PE events, which is maybe driven by an elevated overall mortality of male PE patients in the nationwide in-patient sample.

Obesity influences the survival of several acute cardiovascular diseases (CVD), beneficially indicating an obesity survival paradox in these CVD. Despite the adverse effect of obesity on most cardiovascular risk factors as well as on the prevalence of most CVD, studies have reported an obesity paradox leading to a better prognosis for obese patients with CVD compared to their leaner counterparts [65,66,67]. A possible explanation is that obesity may protect against malnutrition and energy waste during the acute phase of these critical, life-threatening diseases [68,69,70].

Similarly, although arterial hypertension is an important risk factor for the development of ischemic stroke as well as myocardial infarction [71], it is well known, that low blood pressure in these acute manifestations and conditions of ischemic stroke [72,73] and myocardial infarction [74] is strongly associated with increased mortality. Consistently in acute PE, hypotension is accompanied by increased short-term mortality [56]. Therefore, our results lead to the hypothesis that an existing arterial hypertension may modify the drop in blood pressure response or that a higher base level in systolic blood pressure is responsible for the better survival of patients with arterial hypertension during the acute phase.

The main interest regarding the use of systemic thrombolysis was the hypothesis that systemic thrombolysis should be particularly beneficial in these critically ill patients with both life-threatening diseases (PE and ischemic stroke), in which systemic thrombolysis is recommended for selected patients [56,73,75]. Our data support the hypothesis that an uncritically and unselected use of systemic thrombolysis treatment seems unreasonable, whereas the use of systemic thrombolysis according to stroke and PE guidelines was beneficial for selected patients. This recommendation was confirmed by our study showing that the use of systemic thrombolysis was harmful when administered in PE patients with ischemic stroke regardless of hemodynamic status, but in PE patients with additionally diagnosed stroke, who had to undergo CPR, systemic thrombolysis led to a better survival. In this context, a 0.5-fold decreased short-term risk of dying driven by the administration of systemic thrombolysis in PE patients with ischemic stroke, who underwent CPR, is an important finding because the benefit regarding systemic thrombolysis seems to be larger than the reported benefit of systemic thrombolysis in PE patients with CPR in other studies [3] and supports the recommendations of the guidelines [56,73,75].

## 5. Limitations

In this context, some main limitations of this study must be considered: Due to the nature of an ICD- and OPS-code-based study analysis for hospitalized patients, under-reporting and under-coding are possible and may be biasing factors. In addition, data on concomitant medications other than systemic thrombolysis, such as heparins or vitamin K antagonists, NOACs, or laboratory markers are not available in the German nationwide in-patient sample. In addition, no follow-up evaluation is available since data are only limited to the timeframe of the in-hospital course. Another key limitation represents the specific focus on outcome parameters such as in-hospital mortality, as we had no opportunity to include additional information about the functional status of the patients, especially the patients with ischemic stroke, which information would be valuable. In this context, for example, several studies have outlined that for stroke patients, the survival rate between both genders was similar, but the functional outcome was less favorable in women [7,61,62,63,64]. However, our study results are partly in accordance with the current recommendations that systemic thrombolysis is beneficial for selected patients with PE [56] and ischemic stroke [73,76].

## 6. Conclusions

Ischemic stroke had a substantially negative impact on the survival of PE patients. The combination of DVT and heart septal defect was the strongest risk factor for ischemic stroke in PE patients. Further predictors were older age, surgery, arterial hypertension, diabetes mellitus type 2, and AF. Age, cancer, chronic lung diseases, renal insufficiency, coagulation abnormalities, AF, and diabetes mellitus type 2 were independent risk factors for increased in-hospital death in PE patients with ischemic stroke. Systemic thrombolysis was associated with PE patients with ischemic stroke, who underwent CPR.

## Figures and Tables

**Table 2:** Factors associating with ischemic stroke event in PE patients (n = 346,586; among these, 6704 [1.9%] patients were additionally diagnosed with ischemic stroke).

	Uni-Variate Regression Model		Multi-Variate Regression Model	
Parameters	OR (95%CI)	*p*-Value	OR (95%CI)	*p*-Value
Age	1.009 (1.007–1.011)	<0.001	1.005 (1.003–1.007)	<0.001
Gender (Females)	1.014 (0.966–1.065)	0.564	1.037 (0.986–1.091)	0.158
Obesity	1.000 (0.918–1.090)	0.996	0.775 (0.708–0.848)	<0.001
**VTE risk factors**				
Surgery during in-hospital stay	2.505 (2.374–2.643)	<0.001	1.686 (1.590–1.789)	<0.001
Cancer	0.796 (0.746–0.849)	<0.001	0.654 (0.611–0.699)	<0.001
**Comorbidities**				
Chronic (left) heart failure	1.122 (1.060–1.188)	<0.001	0.716 (0.672–0.763)	<0.001
Chronic lung disease	1.240 (1.170–1.314)	<0.001	1.031 (0.970–1.095)	0.330
Arterial hypertension	1.456 (1.387–1.528)	<0.001	1.259 (1.196–1.325)	<0.001
Renal insufficiency	1.183 (1.118–1.251)	<0.001	0.789 (0.742–0.840)	<0.001
Diabetes mellitus (type 2 diabetes)	1.486 (1.405–1.571)	<0.001	1.233 (1.114–1.364)	<0.001
Coagulation abnormalities	1.775 (1.661–1.898)	<0.001	1.018 (0.946–1.096)	0.632
Atrial fibrillation/flutter	2.523 (2.394–2.660)	<0.001	2.060 (1.943–2.183)	<0.001
Combination of DVT and heart septal defect	30.060 (25.431–35.532)	<0.001	24.714 (20.693–29.517)	<0.001
**Risk stratification parameters**				
Tachycardia	1.895 (1.656–2.169)	<0.001	1.184 (1.028–1.364)	0.019
Shock	1.982 (1.807–2.173)	<0.001	1.233 (1.114–1.364)	<0.001
Myocardial injury	2.741 (2.470–3.043)	<0.001	1.996 (1.789–2.226)	<0.001
RV dysfunction	1.125 (1.067–1.186)	<0.001	1.077 (1.019–1.139)	0.009

The multi-variable regression model was calculated with all variables in one model (additionally adjusted for length of in-hospital stay and rheumatic diseases [comprised rheumatoid arthritis, Wegener’s granulomatosis, and systemic lupus erythematosus] and in-hospital stay). Abbreviations: RV indicates right ventricular. *p*-values of <0.05 mark statistically significant values.

**Table 3:** Baseline characteristics, medical history, and presentation of the 6704 PE patients with additional diagnosis of ischemic stroke stratified according in-hospital mortality.

Parameters	PE Patients with Stroke, Who Died In-Hospital (n = 1938; 28.9%)	PE Patients with Stroke, Who Were Discharged Alive (n = 4766; 71.1%)	*p*-Value
Age	75.0 (65.0–82.0)	73.0 (62.0–80.0)	<0.001
Gender (females)	1018 (52.5%)	2580 (54.1%)	0.232
In-hospital stay (days)	10 (4–20)	20 (12–33)	<0.001
Obesity	130 (6.7%)	449 (9.4%)	<0.001
**VTE risk factors**			
Surgery during in-hospital stay	1196 (24.8%)	3636 (75.2%)	<0.001
Cancer	419 (21.6%)	694 (14.6%)	<0.001
**Comorbidities**			
Chronic (left) heart failure	524 (27.0%)	1073 (22.5%)	<0.001
Chronic lung disease	471 (24.3%)	1045 (21.9%)	0.035
Arterial hypertension	929 (47.9%)	2698 (56.6%)	<0.001
Renal insufficiency	625 (32.2%)	1017 (21.3%)	<0.001
Diabetes mellitus (type 2 diabetes)	525 (27.1%)	1185 (24.9%)	0.058
Deep venous thrombosis or thrombophlebitis	322 (16.6%)	1283 (26.9%)	<0.001
Coagulation abnormalities	372 (19.2%)	682 (14.3%)	<0.001
Heart septal defect	60 (3.2%)	474 (9.9%)	<0.001
Atrial fibrillation/flutter	652 (33.6%)	1415 (29.7%)	0.001
Combination of DVT and heart septal defect	16 (0.8%)	205 (4.3%)	<0.001
**Risk stratification**			
Tachycardia	85 (4.4%)	141 (3.0%)	0.003
Syncope	24 (1.2%)	88 (1.8%)	0.078
Shock	303 (15.6%)	203 (4.3%)	<0.001
Myocardial injury	167 (8.6%)	226 (4.7%)	<0.001
RV dysfunction	874 (45.1%)	1117 (23.4%)	<0.001

Abbreviations: VTE indicates venous thromboembolism, which comprises the two VTE entities of pulmonary embolism and deep venous thrombosis; RV, right ventricular. *p*-values of <0.05 (two-sided) mark statistically significant values.

**Table 4:** Predictors of in-hospital death in PE patients with additionally diagnosed ischemic stroke (n = 6704; among these, 1938 [28.9%] patients died in-hospital).

	Uni-Variate Regression Model		Multi-Variate Regression Model	
Parameters	OR (95%CI)	*p*-Value	OR (95%CI)	*p*-Value
Age	1.013 (1.009–1.017)	<0.001	1.011 (1.006–1.016)	<0.001
Gender (females)	0.938 (0.843–1.042)	0.232	0.858 (0.760–0.969)	0.014
Obesity	0.691 (0.564–0.847)	<0.001	0.772 (0.613–0.973)	0.028
**VTE risk factors**				
Surgery during in-hospital stay	0.501 (0.447–0.561)	<0.001	0.760 (0.663–0.873)	<0.001
Cancer	1.618 (1.414–1.852)	<0.001	2.275 (1.947–2.659)	<0.001
**Comorbidities**				
Chronic (left) heart failure	1.275 (1.130–1.440)	<0.001	0.941 (0.814–1.087)	0.409
Chronic lung diseases	1.143 (1.009–1.295)	0.035	1.224 (1.063–1.411)	0.005
Arterial hypertension	0.706 (0.635–0.785)	<0.001	0.722 (0.640–0.816)	<0.001
Renal insufficiency	1.755 (1.560–1.974)	<0.001	1.558 (1.356–1.790)	<0.001
Diabetes mellitus (type 2 diabetes)	1.123 (0.996–1.266)	<0.001	1.265 (1.103–1.452)	0.001
Coagulation abnormalities	1.423 (1.238–1.635)	<0.001	1.676 (1.415–1.986)	<0.001
Atrial fibrillation/flutter	1.201 (1.073–1.344)	0.001	1.238 (1.083–1.414)	0.002
Combination of DVT and heart septal defect	0.185 (0.111–0.309)	<0.001	0.178 (0.101–0.312)	<0.001
**Risk stratification parameters**				
Tachycardia	1.505 (1.143–1.980)	0.004	1.374 (0.997–1.894)	0.052
Shock	4.166 (3.457–5.020)	<0.001	4.218 (3.362–5.293)	<0.001
Myocardial injury	1.894 (1.539–2.331)	<0.001	1.687 (1.333–2.135)	<0.001
RV dysfunction	2.683 (2.400–3.001)	<0.001	2.518 (2.220–2.857)	<0.001

The multi-variate regression model was calculated with all variables in one model (additionally adjusted for length of in-hospital stay and rheumatic diseases [comprised rheumatoid arthritis, Wegener’s granulomatosis, and systemic lupus erythematosus] and in-hospital stay). Abbreviations: RV indicates right ventricular. *p*-values of <0.05 mark statistically significant values.

## Data Availability

All codes used in this study are publicly available online. The data used in this study are sensitive due to individual patient-level data and will not be made publicly available. The data is available at the Federal Statistical Office of Germany (Statistisches Bundesamt, DEStatis) (source: RDC of the Federal Statistical Office and the Statistical Offices of the federal states, DRG Statistics 2011–2014, and own calculations).
